# THE GIFT OF MUSIC: AN INTERGENERATIONAL CAMPUS-COMMUNITY PARTNERSHIP

**DOI:** 10.1093/geroni/igac059.3030

**Published:** 2022-12-20

**Authors:** Whitney Nesser, Scott Buchanan

**Affiliations:** Indiana State University, Terre Haute, Indiana, United States; Indiana State University, Terre Haute, Indiana, United States

## Abstract

Intergenerational programs bring individuals together across a continuum of age to share experiences. This study was designed to engage university music students with residents at a senior living community. University music student groups performed monthly at a local senior living community and completed pre- and post- performance evaluations to assess performance expectations, level of interest in the performance, perception of factors determining performance success, and perception of performance importance for the senior residents. A total of 24 students participated in one of three musical ensembles (Choir=11; Flutes=5; Steel Drums=8) during the months of February, March, and April 2022. Across all three ensembles, 50% of the students had never performed for residents at a senior living community. On a scale of 1–5, with 1 being “Not at all” and 5 being “Very much”, 21 students indicated a “4” or “5” as to the importance of the performance for the residents whereas 15 students indicated a “4” or “5” in response to the importance of the performance for themselves. Most students reported looking forward to the performance, and following the performance indicated it had been a success. Factors identified as determining performance success included comments such as: “How much the audience enjoyed themselves!” and “Performers and audience enjoyed the performance. We would love to come back!”. Our findings suggest that performing live music in an intergenerational campus-community setting is beneficial not only for students but also for senior living facility residents.

